# Evaluation of Morphologic Dimensions of Humulus Appendix of Pterygoid Plate Using Cone Beam Computed Tomography (CBCT) in Ahvazian Patients, Iran -

**DOI:** 10.31661/gmj.vi.3901

**Published:** 2025-12-16

**Authors:** Zahra Saadi, Sanaz Sharifi Shushtari, Alireza Hashemi Ashtiani, Ali Tayebi

**Affiliations:** ^1^ Department of Oral and Maxillofacial Radiology, School of Dentistry, Ahvaz Jundishapur University of Medical Sciences, Ahvaz, Iran; ^2^ Department of Prosthdontics, School of Dentistry, Ahvaz Jundishapur University of Medical Sciences, Ahvaz, Iran

**Keywords:** Cone Beam Computed Tomography, Inclination, Axial, Coronal, Length, Width, Humulus of Pterygoid Appendage

## Abstract

**Background:**

Today, CBCT has found a special place in dentistry due to the high quality and accuracy of images and providing information, and its use is increasing. With its help, we can examine many parts of the anatomy that are difficult to evaluate. The purpose of this study is to investigate the morphological dimensions of the humulus of the pterygoid appendage using cone beam computed tomography (CBCT).

**Materials and Methods:**

In this retrospective study, the statistical population was the imaging reccords of patients who referred to the radiology department of Ahvaz Dental School for CBCT of the upper jaw, whose values were stored in the NNT software. The size of the humulus (length and width) and its slope in the coronal and axial sections of the images were evaluated by two oral and maxillofacial radiologists. The results were analyzed using SPSS software version 22.

**Results:**

Eighty pterygoid hamuli from 38 males and 42 females (age 26–64 years, mean 43.18 ± 11.57) were analyzed. No significant gender differences were observed in length (P=0.096), width (P=0.300), axial angle (P=0.067), or coronal angle (P=0.102). Age-related comparisons revealed significant variation: hamular length and width increased in the 31–40 and 51 year groups versus 30 years (P=0.022–0.031), axial angle was higher in 31–40 and 51 year groups (P=0.003–0.006), and coronal angle increased in 31–40 and 51 year groups (P=0.047–0.049).

**Conclusion:**

These findings indicate gradual morphometric changes with age, independent of gender. The length of PH increases with age and then decreases. While the width increases with age. There was no significant difference between length and width measurements in men and women. These findings help to diagnose vague pains in the oropharynx region related to the altered morphology of PH.

## Introduction

The sphenoid bone serves as a reference structure for connecting almost all other skull bones, positioned at the base of the skull in front of the temporal and basilar parts of the occipital region. In addition to this structural significance, this bone is divided into a central or median part, two large wings, and two small wings extending outward from the body, along with two pterygoid processes beneath it, formed by a lateral plate and an internal plate [1]. Furthermore, the Pterygoid Hamulus (PH) is a bony structure located at the bottom of the internal plate of the sphenoid bone [2]. Due to this specific anatomical placement, its location at the base of the skull and the variety of anatomical structures whose connections are situated on the surface of the pterygoid hamulus are of significant functional importance [3]. More specifically, the hamulus is a small hook-like projection at the top and bottom of the internal pterygoid plate. In fact, the location, length, and width of this projection play a critical role in the function of several muscles, such as the tensor veli palatini, palatopharyngeal, and the superior part of the pharyngeal muscle, among others. As a result, these muscles separate the oral cavity from the nasal cavity during swallowing. Therefore, the position and length of the hamulus are vital for the efficiency of these muscles [4][5]. Interestingly, according to several studies on the position and morphology of the PH in different populations, it was found that the length of the PH ranges from 4.9 mm to 7.2 mm [6][7].

However, an increase in the length of the hamulus can cause complex symptoms in the soft palate and throat, such as severe pain in the throat and palate, which may be limited or spread to the ear and temporomandibular joint. Moreover, this pain may occur spontaneously or be triggered by touch or drinking fluids. Because of this, due to the muscular attachments of this projection, it can cause significant problems in this area and mimic symptoms of other diseases. Additionally, it can also cause issues during prosthetic molding. Consequently, awareness of the morphology of this structure and accurate interpretation of radiographs may provide valuable information for diagnosing pain in the oral cavity [8][9][10][11][12][13][14][15][16]. Regarding diagnostic approaches, diagnosis of the hamulus in this region through radiographs such as cephalometry, waters, and submentovertex views is possible, but due to the limitations of conventional radiographs, its diagnosis is challenging. To overcome these limitations, CT scan images may allow for the accurate evaluation of cranial and facial structures by overcoming the problems of conventional radiography, although it, unfortunately, imposes high doses and costs on the patient. Fortunately, recently, Cone Beam Computed Tomography (CBCT) has provided 3D images at lower doses and costs for the physician, paving the way for more accurate assessments [15][16][17]. Furthermore, with the introduction of new programs in CBCT, this method is continually improving [18]. Since CBCT is a true 3D imaging technique, isotropic spatial resolution with very small voxel sizes is achievable [19]. Therefore, given the high precision of CBCT, we aimed to conduct a study to examine the morphological dimensions of the pterygoid hamulus using CBCT in patients visiting the Dental School of Ahvaz.

## Materials and Methods

This study is retrospective study conducted on imaging records of patients who visited the Radiology Department of the Dental School of Ahvaz for maxillary CBCT scans between 2022 to 2024. Since this study was conducted on archived images from patients visiting the Radiology Department for maxillary CBCT at the Dental School of Ahvaz, there was no need to obtain consent forms, and no special ethical considerations were required. The sample size was determined based on the results of Romoozi et al. [20], which investigated the morphology of the pterygoid hamulus using CBCT. Using the Med-Cal statistical software with 1% accuracy and a 95% confidence interval, 80 cases were selected. Since the study was based on CBCT images from the Radiology Department of the Dental School of Ahvaz, the samples were conveniently selected.

The images embedded on NNT software were examined by two oral and maxillofacial radiology specialists on a 14-inch LED flat monitor (ASUS, 1920 × 1080 resolution) in a semi-dark room using the NNT software. The observers were able to view the images in both axial and coronal planes. If necessary, contrast and brightness were adjusted by the observers. The size of the hamulus (length and width) at its thickest and longest point in the coronal sections was measured using a digital ruler in the NNT software. Additionally, the inclination of the projection in the coronal plane relative to the base of the sphenoid at the separation of the pterygoid plates, as well as in the axial plane, was measured at the same location in the most distinct section. The length and width of the pterygoid hamulus at its longest and thickest point in the coronal sections, as well as the sagittal and coronal plane inclinations of the pterygoid hamulus, were measured by both observers using a digital ruler. The results obtained were then provided to a statistical expert for further analysis. The data in this study were analyzed using SPSS software version 22 (SPSS 22 for Windows, SPSS Inc., Chicago, IL). Initially, descriptive statistics methods including frequency distribution tables, charts, and measures of central tendency and dispersion were used to describe the variables under study. To test the normality of the distribution, the Shapiro-Wilk test was applied. Given that the data followed a normal distribution, ANOVA and T-test were used. A significance level of P≤0.05 was considered for all statistical tests.

## Results

**Table:** Table1. Hamular Length, Width, Axial and Corneal Angels of Pterygoid Hamuli Stratified by Age and Gender

Parameter		Groups		N	Mean	SD/%	P
		Total		80	7.43	1.3224	—
	Gender		Males	38	7.613	1.5441	0.096
			Females	42	7.264	1.0664	
**Length (mm) **			<30 years	10	7.08	1.1826	0.024
	Age group		31-40 years	30	7.803	1.5244*	
			41-50 years	18	7.044	1.0916	
			>51 years	22	7.395	1.1459	
		Total		80	1.664	0.4649	—
	Gender		Males	38	1.704	0.4614	0.3
			Females	42	1.627	0.4678	
**Width (mm) **			<30 years	10	1.395	0.3187	0.015
	Age group		31-40 years	30	1.628	0.5089**	
			41-50 years	18	1.761	0.3383	
			>51 years	22	1.755	0.5041***	
		Total		80	51.439	12.9754	—
	Gender		Males	38	53.416	13.1131	0.067
			Females	42	49.65	12.6619	
**Axial Angle (°) **			<30 years	10	42.86	11.0289	0.002	
	Age group		31-40 years	30	54.14	12.4462 †	
			41-50 years	18	48.583	12.2473	
			>51 years	22	53.991	13.276 ††	
		Total		80	106.053	8.1461	—
	Gender		Males	38	107.161	9.4803	0.102
			Females	42	105.05	6.6156	
**Coronal Angle (°) **			<30 years	10	103.19	8.115	0.038
	Age group		31-40 years	30	106.69	6.9476 ‡	
			41-50 years	18	103.944	10.2407	
			>51 years	22	108.209	7.1991 ‡ ‡	

^*^ Post-hoc Tukey test of width: 31–41 years vs <30 years, p = 0.031. ^**^ Post-hoc Tukey test of length: 31–41 years vs <30 years, p = 0.022. ^***^ Post-hoc Tukey test of length: >51 years vs <30 years, p = 0.020. † Post-hoc Tukey test of axial angle: 31–41 years vs <30 years, p = 0.003. †† Post-hoc Tukey test of axial angle: >51 years vs 41–50 years, p = 0.006 ‡ Post-hoc Tukey test of corneal angle: 31–41 years vs <30 years, p = 0.047. ‡‡ Post-hoc Tukey test of corneal angle: >51 years vs <30 years, p = 0.049.

**Figure-1:**
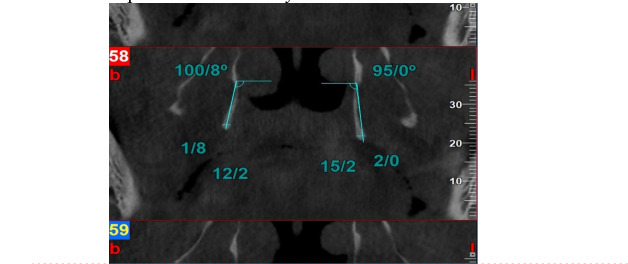
Figure 1. A sample of CBCT images taken in the coronal section

**Figure-2:**
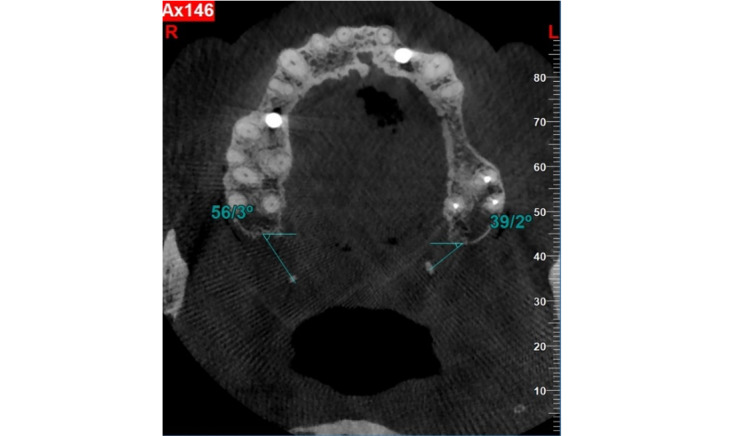
Figure 2. A sample of CBCT images taken in the axial section

A total of 80 pterygoid hamuli were analyzed in this morphometric study, comprising 38 males (47.5%) and 42 females (52.5%), with ages ranging from 26 to 64 years (mean=43.18 ± 11.57 years). The average age of male participants was 43.42 ± 9.90 years, while that of females was 42.95 ± 12.95 years, showing no significant difference (P=0.851). Participants were categorized into four age groups: <30 years (12.5%), 31-40 years (37.5%), 41-50 years (22.5%), and >51 years (27.5%), providing balanced representation across adult age ranges.

Regarding pterygoid hamulus length, the overall range was 5.0-11.8 mm with a mean of 7.43 ± 1.32 mm. Males exhibited slightly greater lengths (7.61 ± 1.54 mm) compared to females (7.26 ± 1.07 mm); however, the difference was statistically insignificant (t=1.676, P=0.096, df=158). When analyzed by age, the length demonstrated a significant difference among age groups (ANOVA: F=3.221, P=0.024). Post-hoc testing revealed that the 31-40 year group (mean=7.80 mm) had significantly longer hamuli than the <30 year group (mean=7.08 mm; P=0.031, mean difference=0.759, 95% CI=0.050-1.468). No other pairwise differences were significant.

For hamular width, the total mean was 1.66 ± 0.46 mm, ranging from 1.0 to 3.2 mm. Males showed a mean width of 1.70 ± 0.46 mm, and females 1.63 ± 0.47 mm; the difference was statistically nonsignificant (t=1.041, P=0.300, df=158). In contrast, age-based comparison indicated significance (ANOVA: F=3.597, P=0.015). The <30 year group had the smallest mean width (1.40 ± 0.32 mm), whereas older groups displayed broader structures (≥ 1.75 mm). Post-hoc analysis confirmed significant differences between <30 years and both 31-40 years (P=0.022) and >51 years (P=0.020), implying gradual thickening of the hamulus with age.

The axial angle of the pterygoid hamulus varied from 23.6° to 80.0°, with a total mean of 51.44 ± 12.98°. Males demonstrated a slightly greater mean (53.42 ± 13.11°) than females (49.65 ± 12.66°), but the difference was not statistically significant (t=1.847, P=0.067). However, ANOVA revealed significant variation across age groups (F=5.333, P=0.002). The youngest group (<30 years) had the smallest mean axial angle (42.86° ± 11.03°), significantly lower than both the 31-40 year (54.14° ± 12.45°) and >51 year (53.99° ± 13.28°) groups (P=0.003 and P=0.006, respectively).

The coronal angle ranged between 89.4° and 130.1°, with an overall mean of 106.05 ± 8.15°. The mean for males (107.16 ± 9.48°) was marginally higher than for females (105.05 ± 6.62°), but the difference was not statistically significant (t=1.645, P=0.102, df=158). In age-wise comparison, ANOVA detected a significant difference (F=2.876, P=0.038). Post-hoc tests indicated that the coronal angle was significantly greater in the 31-40 year (106.69 ± 6.95°) and >51 year (108.21 ± 7.20°) groups compared to the 41-50 year group (103.94 ± 10.24°; P=0.047 and P=0.049, respectively).

## Discussion

Our study evaluated the morphologic dimensions of the pterygoid hamulus (PH) using CBCT in an Ahvazian population, focusing on length, width, and angular inclinations in coronal and axial planes, and examining age- and gender-related variations. We observed that PH length and width generally increased with age, while no significant gender differences were noted. This diverges from Iranian studies like Mahdavi and Hafezi [21], who found no age-related variations in women aged 20-40. Similar to Khoubivand et al. [22], our study confirmed lateral and posterior inclinations of PH, suggesting that angular orientations may be more conserved than linear dimensions, potentially reflecting functional adaptations in masticatory biomechanics.

When comparing morphological trends across studies, subtle differences emerge. Our results showed incremental increases in PH length in the 31-40 and >51 age groups, suggesting a non-linear growth pattern. In contrast, Sivadas et al. [23] and Mehra et al. [24] reported a more continuous increase in both length and width with age. Gender effects were inconsistent: our Ahvazian cohort showed no significant sex differences, whereas Mehra et al. and Sivadas et al. reported longer and wider PHs in males, highlighting regional or ethnic anatomical variability. Although Sivadas et al. noted different PH shapes, including slender and triangular forms, our study did not explicitly classify shapes; however, observed gradual morphological changes suggest that shape variability may accompany dimensional growth, which could be clinically relevant for oropharyngeal pain syndromes.

Consistent with our findings, Krmpotić-Nemanić et al. [25] in Croatia reported that older patients had shorter PH compared to younger patients. Similarly, Orhan et al. [10] in Turkey reached the same conclusion. These consistent results across populations reinforce the strength of our findings. However, Komarnitki et al. [26] and Putz and Kroyer [6] reported PH length increases with age, with Putz and Kroyer noting stability after adulthood. Variations may reflect population and methodological differences.

In our study, PH width increased with age, and reduction after 50 years was not significant compared to the 40-50-year-old group. This aligns with Nerkar et al. [27], while Mehra et al. [22] reported a decrease followed by a non-significant increase, and Romoozi et al. [20] found a decrease with age. Orhan et al. [10] observed no significant difference in width between two age groups, similar to our finding that width differences above 55 years were not significant. These differences likely reflect population and methodological variations.

Our study revealed no significant gender differences in PH length or width, consistent with Orhan et al. [10[, Nerkar et al. [27], and Romoozi et al. [20]. Conversely, Mehra et al. [22] and Komarnitki et al. (26) reported significant gender differences, with males having greater PH dimensions. Discrepancies may be attributed to population and methodological differences.

A significant relationship was observed between age groups and coronal plane PH inclination, likely reflecting morphological changes with age, but no gender-based differences were noted. Romoozi et al. [20] reported similar lack of gender differences but did not find significant age-related differences, likely due to population differences.

The clinical and diagnostic implications of these findings are notable. Our study, consistent with Orhan et al. [10] and Khoubivand et al. [22], emphasizes CBCT’s role in evaluating PH morphology to interpret idiopathic oropharyngeal pain. Variations across populations highlight the need for region-specific reference data, as differences in angular inclinations, age-related growth patterns, and gender influences suggest standardized diagnostic criteria should consider demographic context. Additionally, symmetry and bilateral comparisons reported in Alalawi et al. [28] reinforce that surgical planning in the pterygomaxillary region must account for minor yet clinically significant morphometric asymmetries, underscoring the contribution of our study to understanding PH anatomy in the Iranian population and its potential forensic, surgical, and diagnostic applications.

## Conclusion

In the present study, it was found that the average length of the hamulus was 7.430 ± 0.1045 mm, the average width was 1.664 ± 0.0368 mm, the average axial slope was 51.439 ± 1.0258°, and the average coronal slope was 106.053 ± 0.6440°. A significant relationship was observed between most age groups and the axial and coronal slopes, as well as the length and width of the pterygoid hamulus (PH). However, no significant relationship was found based on gender. The length of the PH increases with age and then decreases, while the width increases with age, but no significant difference is observed in its decrease. There was no significant difference in length and width measurements between males and females. These findings can assist in diagnosing ambiguous pain in the oropharyngeal region related to the altered morphology of the PH.

## Conflict of Interest

None.
